# Distinct cytokine profiles in late pregnancy in Ugandan people with HIV

**DOI:** 10.1038/s41598-024-61764-2

**Published:** 2024-05-14

**Authors:** Lisa M. Bebell, Joseph Ngonzi, Audrey Butler, Elias Kumbakumba, Julian Adong, Carolin Loos, Adeline A. Boatin, Ingrid V. Bassett, Mark J. Siedner, Paige L. Williams, Heather Mattie, Bethany Hedt-Gauthier, Katharine F. B. Correia, Erin Lake, Galit Alter

**Affiliations:** 1 Medical Practice Evaluation Center and Center for Global Health, Massachusetts General Hospital Division of Infectious Diseases, GRJ-504, 55 Fruit St, Boston, MA 02114 USA; 2 Department of Obstetrics and Gynaecology, Mbarara University of Science and Technology, Mbarara, Uganda; 3 State University of New York Upstate Medical University, Syracuse, NY USA; 4 Department of Paediatrics and Child Health, Mbarara University of Science and Technology, Mbarara, Uganda; 5 Ragon Institute of MGH, MIT and Harvard, Cambridge, MA USA; 6 Department of Obstetrics and Gynecology and Center for Global Health, Massachusetts General Hospital, Boston, MA USA; 7 Division of Infectious Diseases and Medical Practice Evaluation Center, Massachusetts General Hospital, Boston, MA USA; 8 Department of Biostatistics, Harvard T.H. Chan School of Public Health, Boston, MA USA; 9 Department of Global Health and Social Medicine, Harvard Medical School, Boston, MA USA; 10 Department of Mathematics and Statistics, Amherst College, Amherst, MA USA

**Keywords:** Epidemiology, Translational research

## Abstract

During pregnancy, multiple immune regulatory mechanisms establish an immune-tolerant environment for the allogeneic fetus, including cellular signals called cytokines that modify immune responses. However, the impact of maternal HIV infection on these responses is incompletely characterized. We analyzed paired maternal and umbilical cord plasma collected during labor from 147 people with HIV taking antiretroviral therapy and 142 HIV-uninfected comparators. Though cytokine concentrations were overall similar between groups, using Partial Least Squares Discriminant Analysis we identified distinct cytokine profiles in each group, driven by higher IL-5 and lower IL-8 and MIP-1α levels in pregnant people with HIV and higher RANTES and E-selectin in HIV-unexposed umbilical cord plasma (*P*-value < 0.01). Furthermore, maternal RANTES, SDF-α, gro $\mathrm{\alpha }$-KC, IL-6, and IP-10 levels differed significantly by HIV serostatus (*P* < 0.01). Although global maternal and umbilical cord cytokine profiles differed significantly (*P* < 0.01), umbilical cord plasma profiles were similar by maternal HIV serostatus. We demonstrate that HIV infection is associated with a distinct maternal plasma cytokine profile which is not transferred across the placenta, indicating a placental role in coordinating local inflammatory response. Furthermore, maternal cytokine profiles in people with HIV suggest an incomplete shift from Th2 to Th1 immune phenotype at the end of pregnancy.

## Introduction

During pregnancy, multiple immune regulation mechanisms are engaged to create an immune-tolerant environment for the allogeneic fetus. These mechanisms promote fetal growth while also allowing maternal immune defenses to protect the growing fetus from potential pathogens^1^. Immune balance in pregnancy is regulated in part by extraembryonic trophoblast cells that recruit and modulate maternal peripheral blood immune cells. The classical paradigm of immune response in pregnancy is a shift away from a T-helper (Th)1 cellular-mediated immune response, and shift towards a Th2 humoral (antibody)-mediated immune response^2^. Although this paradigm has evolved, this classification allows us to describe the immunoregulatory pathways in pregnancy. This Th1 to Th2 shift in pregnancy results in secretion of anti-inflammatory signals from maternal myeloid and T-cells, called cytokines, that maintain fetal immune tolerance. Without these anti-inflammatory signals, the allogenic fetus could be rejected by the maternal immune system^2^. Thus, the cytokine environment of pregnancy is critically important to maintaining the fine immune balance of pregnancy—protecting the maternal–fetal dyad from infections while also preventing fetal immune rejection^3^. Despite emerging research in this area, the mechanisms and factors regulating immune tolerance and fetal protection in pregnancy are incompletely understood. Furthermore, the impact of chronic maternal infections, including human immunodeficiency virus (HIV), on immune balance in pregnancy is incompletely characterized^2,4^.

Maternal coinfections may alter the in utero inflammatory environment through changes in secretion of cytokines and chemokines (specific cytokines attracting cells to inflamed sites, hereafter all referred to using the umbrella term ‘cytokines’). People with HIV have high levels of inflammation and residual immune dysfunction, even in the setting of effective antiretroviral therapy (ART), disrupting fetal immune homeostasis and altering immune cell function in HIV-exposed children. The potential impact of HIV on the cytokine and chemokine environment in pregnancy, as well as on pregnancy outcomes, has not been fully characterized^5,6^. HIV treatment in reproductive-aged people has led to a marked decline in the incidence of perinatal HIV transmission and over one million HIV-exposed, uninfected (HEU) children born annually. HEU children have a higher risk of severe infections^7–11^ and infection-related hospitalization than HIV-unexposed children^8,12–14^. The causes of increased infection risk and severity in HEU children are largely unknown^15–18^, though immune changes including altered cytokine environment during pregnancy could alter fetal immune development and increase postnatal infection susceptibility. Furthermore, the effects of maternal antiretroviral therapy (ART) in pregnancy are poorly understood. ART may suppress anti-inflammatory molecules (cytokines) that maintain healthy pregnancies and has been associated with increased pregnancy loss in some studies^19–21^.

Understanding how the cytokine environment during pregnancy is altered by maternal HIV infection and ART use is critically important to understanding the origins of adverse birth outcomes in HEU children. In addition, preterm birth, low birth weight and feto-neonatal infectious and inflammatory disorders are more common both in people with HIV and people living in resource-limited settings (RLS)^22–26^. To date, most existing data on the immune and inflammatory environment in pregnancy are derived from people living in resource-rich settings. A clear understanding of the cytokine profiles in maternal and umbilical cord blood in pregnant people living with HIV (PPHIV), and the relationship to maternal comorbidities among people living in resource-limited settings (RLS) could lead to important strategies to optimize the inflammatory environment in pregnancy and improve birth and infectious outcomes among HEU children. To address this gap in knowledge and determine whether, and how cytokine profiles are altered by maternal HIV infection, we comprehensively profiled cytokines and chemokines in a cohort of pregnant people with and without HIV living in Uganda.

## Results

### Demographics and characteristics of PPHIV

We analyzed plasma collected during labor from 147 PPHIV and their paired HIV-exposed umbilical cord blood, and 142 unmatched HIV-uninfected pregnant people and their paired HIV-unexposed umbilical cord blood. The median ages at delivery were 26 and 24 years, respectively (Table 1). Compared to HIV-uninfected women, gravidity was higher in PPHIV, and 26 (25%) of PPHIV with undetectable HIV viral loads were grandmultigravidae compared to 6 (14%) with detectable viral loads. The distribution of gestational age at delivery was similar between groups, with a median of 39 weeks [interquartile range (IQR) 38–40 weeks]. Birthweight was similar between HIV serostatus groups (mean 3.2 kg and standard deviation 0.45 kg for both HIV-uninfected and PPHIV groups). Among PPHIV, all reported taking combination ART at the time of delivery. Fifty-four percent started ART preconception, 28% had a detectable HIV viral load at delivery, and the median CD4 count was 426 cells/mm^3^ (IQR 306, 602). Among those with detectable HIV viral load, median copies/mL were 718 (IQR 265, 12,950). Most (82%) participants reported taking combined tenofovir, efavirenz and either lamivudine or emtricitabine and 18% reported taking another ART combination. Infants were followed through 6 weeks of age and 86% had heelstick HIV DNA test results, all of which were negative.

**Table 1:** Characteristics of 289 pregnant people who delivered at the maternity ward at Mbarara Regional Referral Hospital in Mbarara, Uganda between September 2017 and February 2018.^a^

Characteristic	HIV-uninfected *n* = 142	PPHIV *n* = 147	PPHIV^b^ undetectable^c^ HIV viral load *n* = 105	PPHIV detectable HIV viral load *n* = 42
Age, in years	24 (21, 28)	26 (23, 31)	27 (23, 31)	26 (22, 29)
Gravidity				
Primigravid	46 (32%)	25 (17%)	14 (13%)	11 (26%)
Multigravid (2–4 pregnancies)	75 (53%)	90 (61%)	65 (62%)	25 (60%)
Grandmultigravid (> 4 pregnancies)	21 (15%)	32 (22%)	26 (25%)	6 (14%)
Gestational age, in weeks	39.0 (38.0, 40.3)	39.0 (38.0, 40.0)	39.0 (38.0, 40.2)	39.0 (38.0, 40.0)
Started ART^d^ preconception	–	79 (54%)	65 (62%)	14 (33%)
CD4 T-cell count, in cells/mm^3^	–	426 (306, 602)	440 (320, 617)	389 (227, 563)
Unknown	–	3	2	1
HIV viral load, in copies/mL	–	–	–	718 (265, 12,950)

^a^median (IQR) for continuous, n (%) for categorical, ^b^PPHIV = pregnant person with HIV, ^c^detectable HIV viral load defined as ≥ 50 copies/mL, ^d^ART = combination antiretroviral therapy.

### Cytokine profiles by maternal HIV serostatus and HIV viral load detectability

**Figure 1:**
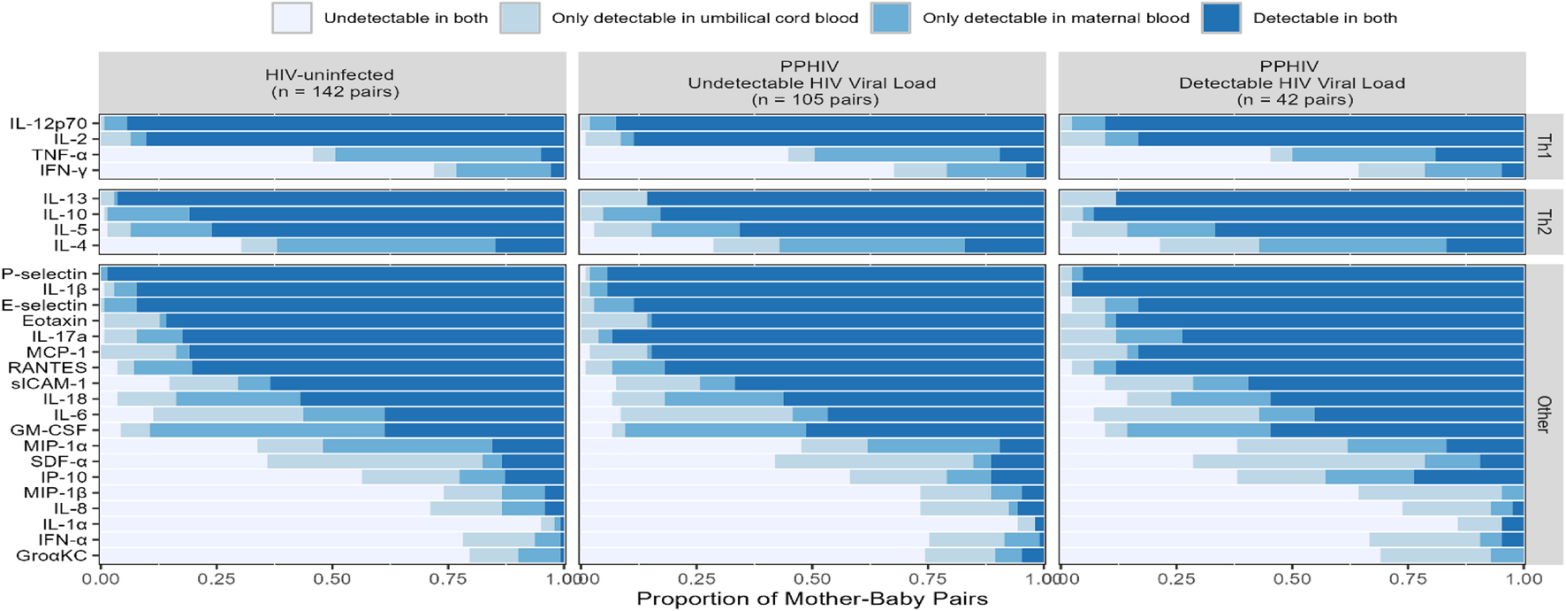
The proportion of mother-baby pairs in which a specific cytokine was detected in neither plasma sample, only one plasma sample, or both paired plasma samples, by maternal HIV serostatus.

The general patterns of cytokine levels were similar between pregnant people without HIV, PPHIV with undetectable HIV viral load, and PPHIV with detectable HIV viral load (Fig. 1). Overall, P-selectin, IL-β, IL-12p70, and IL-13 were the most frequently detected cytokines in both maternal and umbilical cord plasma, detected in over 90% of participants. GM-CSF, IL-4, and TNF-α were more frequently detected in maternal than umbilical cord plasma *(P* < 0.0001), whereas IL-6 and SDF-α were more frequently detected in umbilical cord than maternal plasma *(P* < 0.0001). IL-1α, IFN-α, and Groα KC were the least frequently detected cytokines, detected in less than 30% of samples.

**Figure 2:**
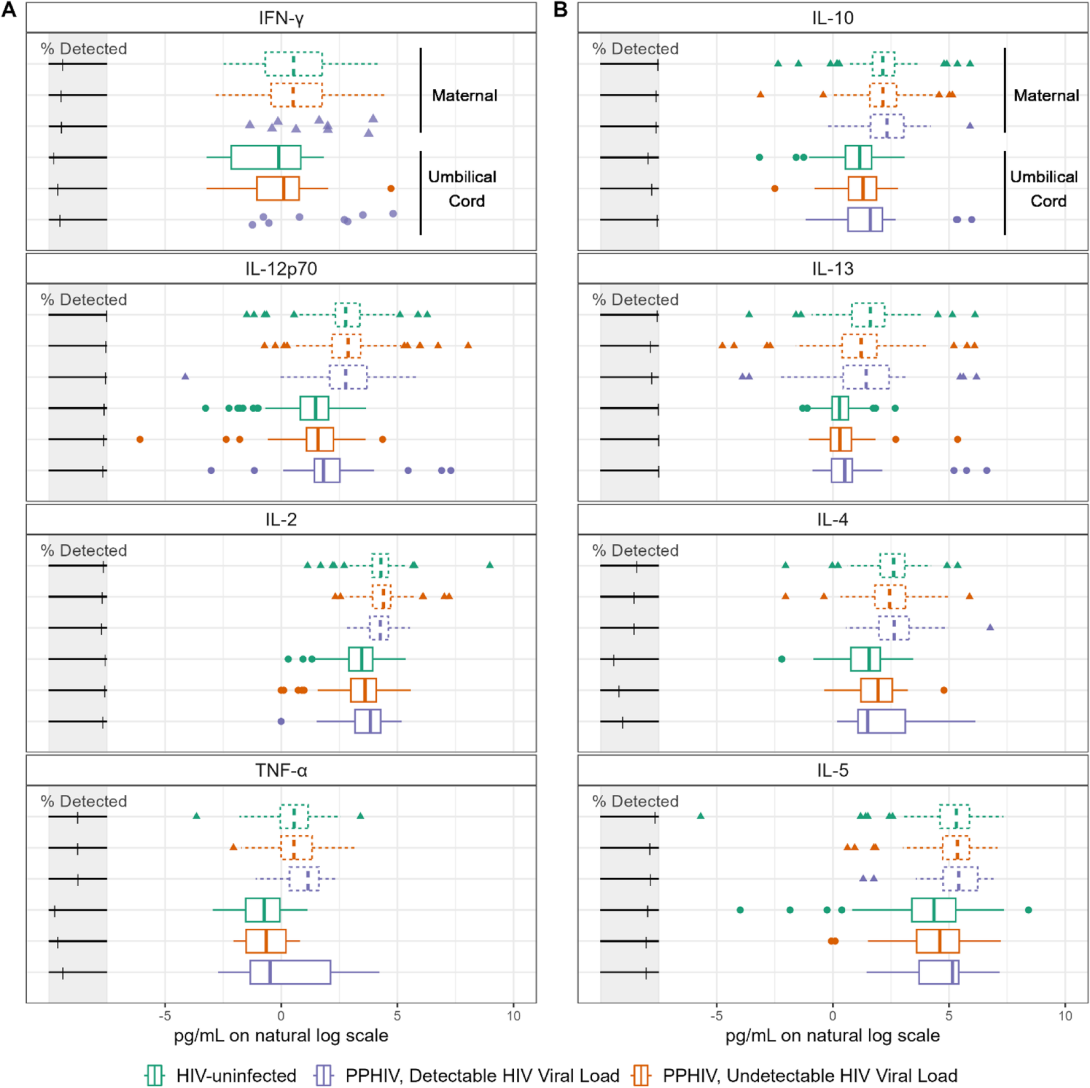
Cytokine concentrations of cytokines classified as Th1 (panel **A**) or Th2 (panel **B**) in maternal and umbilical cord plasma. Cytokine concentrations were natural log transformed.

**Figure 3:**
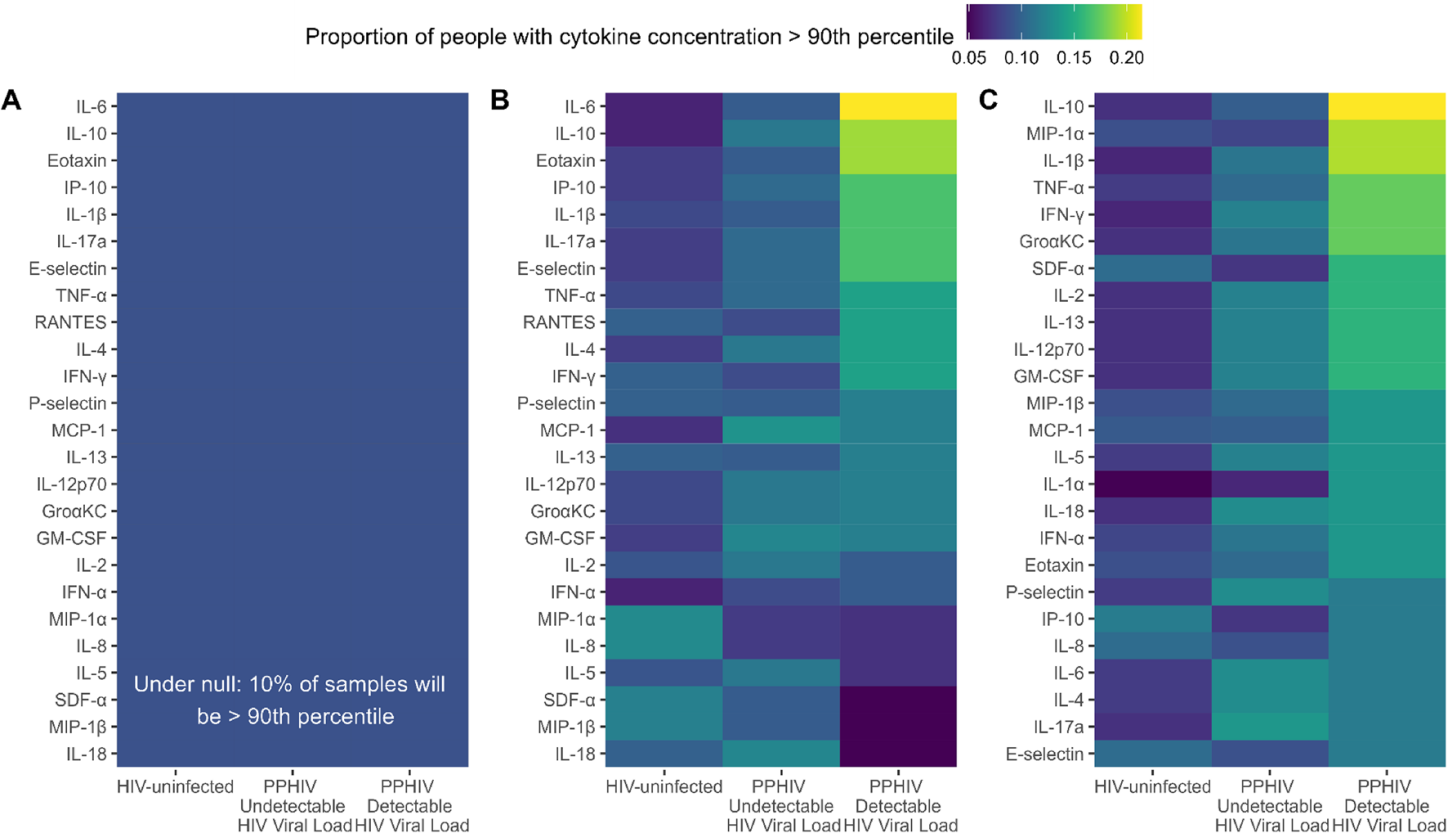
The proportion of samples above 90th percentile for each cytokine concentration, stratified by maternal HIV serostatus, in maternal plasma (**B**) and umbilical cord plasma (**C**). Panel A represents what the figure would look like if HIV serostatus was not associated with extreme cytokine values, where each group would have 10% of samples fall above the 90th percentile.

Overall, the distribution of cytokine concentrations was similar across HIV serostatus and viral load groups in both maternal and umbilical cord plasma (Fig. 2, Supplementary Figs. 2–5). Even after natural log transformation, many of the distributions were skewed, multimodal, and/or had outliers. Though the distribution of concentrations was similar, there were differences in high plasma concentration of specific cytokines by maternal HIV serostatus. If there was no association between HIV serostatus and elevated cytokine concentration, we would expect approximately 10% of each HIV serostatus group to have a cytokine concentration above the 90th percentile (Fig. 3A). However, among maternal plasma samples, 21.4% of PPHIV with detectable HIV viral load had high IL-6 concentration compared to 9.5% of PPHIV with undetectable viral load and 6.3% of HIV-uninfected participants (Fig. 3B), suggesting an association between detectable HIV viral load and detectable maternal plasma IL-6 *(P* = 0.02). PPHIV with detectable HIV viral loads also had higher proportions of elevated IL-10 (19.0%) and eotaxin (19.0%) concentrations *(P* = 0.05 and *P* = 0.11, respectively). Among umbilical cord plasma samples, 23.8% from PPHIV with detectable HIV viral load had a high IL-10 concentration compared to 9.5% of PPHIV with undetectable viral load and 6.3% of HIV-uninfected participants (Fig. 3C), suggesting an association between detectable HIV viral load and detectable umbilical cord plasma IL-10 *(P* = 0.007). Umbilical cord plasma samples from PPHIV with detectable HIV viral loads also had a higher proportion of high MIP-1α (21.4%) and IL-1β (21.4%) concentrations, suggesting associations between detectable HIV viral load and detectable umbilical cord MIP-1α and IL-1β *(P* = 0.05 and *P* = 0.01, respectively).

### Correlations between cytokine concentrations

**Figure 4:**
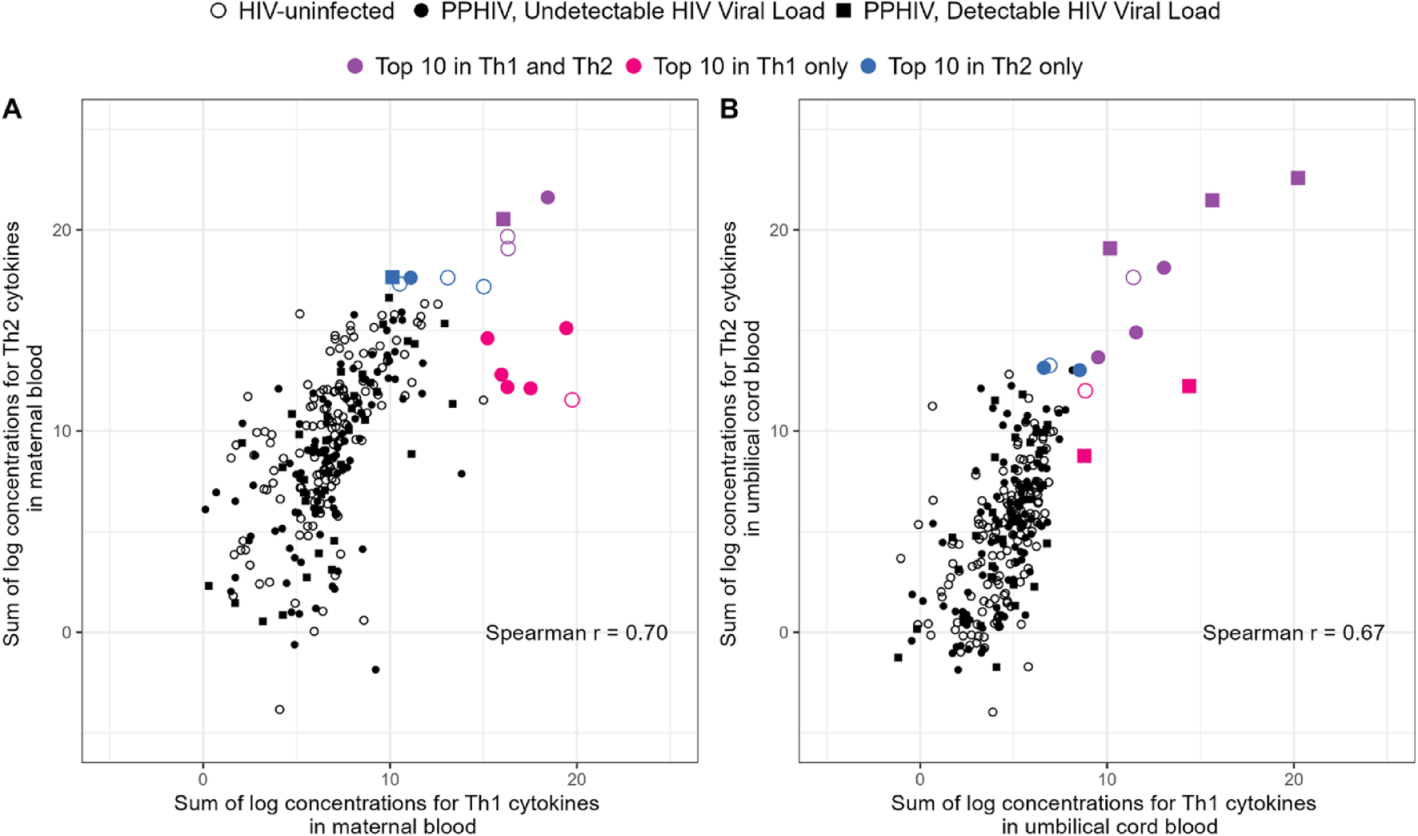
Association between sum of log concentrations for Th1 and Th2 in maternal (panel **A**) and umbilical cord (panel **B**) blood. There is no overlap between the top 10 cases in maternal and umbilical cord blood. The 4 purple points indicate concentrations in the top 10 in both Th1 and Th2, 6 blue points indicate concentrations in the top 10 for Th1 only, and 6 pink points indicate concentrations in the top 10 in Th2 only.

Classically, the inflammatory response in pregnancy has been defined by a shift from Th1 to Th2 phenotype. To further characterize immunoregulatory pathways, cytokines were grouped according to phenotype as Th1 (IFN-γ, IL-12p70, IL-2, TNF-α) and Th2 (IL-10, IL-13, IL-4, IL-5). There was a strong, positive correlation between total summed Th1 cytokine levels and total summed Th2 cytokine levels both in maternal (Spearman r = 0.70, *P* < 0.001) and umbilical cord (Spearman r = 0.67, *P* < 0.001, Fig. 4) plasma. Many participants with the highest Th1 sum also had the highest Th2 sum (Fig. 4). Seven of the ten people with the highest maternal Th1 sum were PPHIV and eight of the ten people with the highest umbilical cord Th1 sum and the highest umbilical cord Th2 sum were PPHIV. In contrast, 51% of the study population were PPHIV.

**Figure 5:**
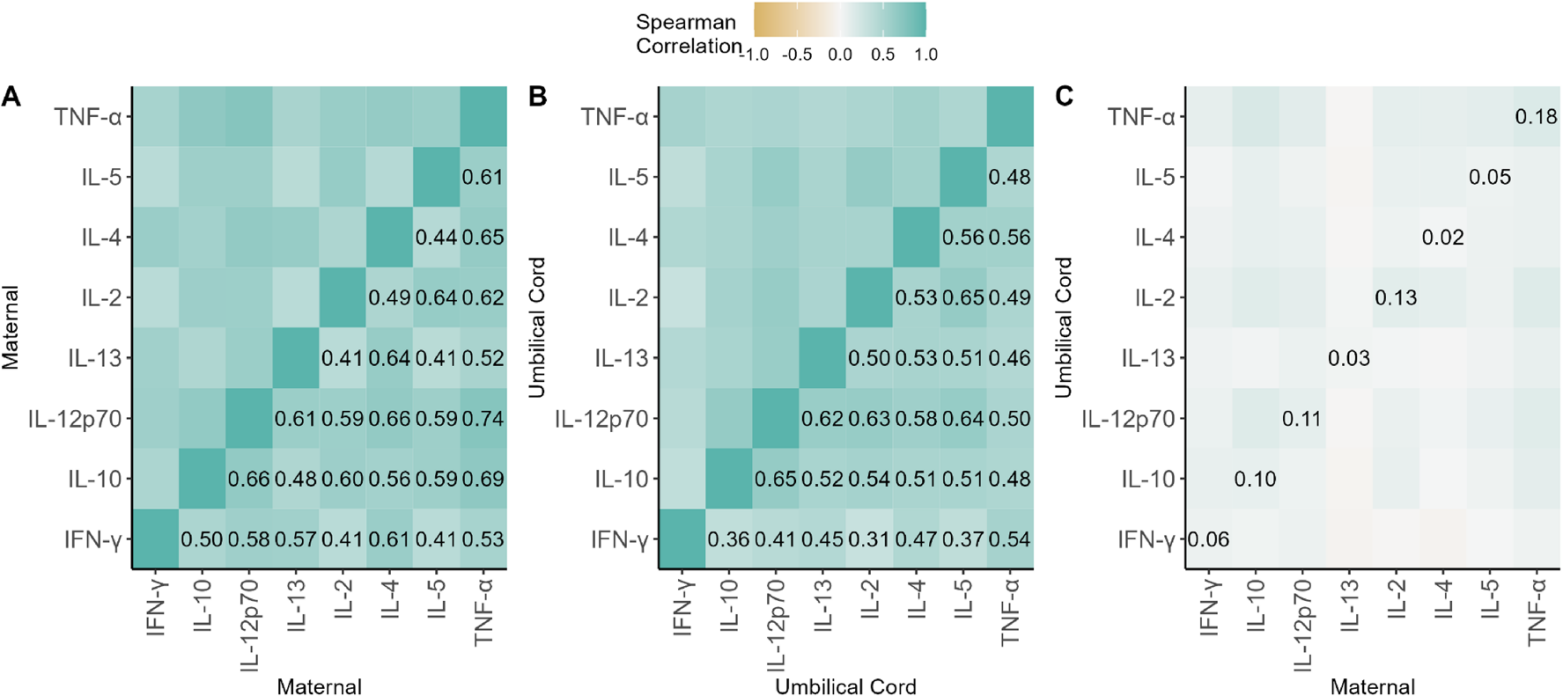
Spearman correlations between cytokine concentrations in maternal plasma (**A**), umbilical cord plasma (**B**), and paired maternal and umbilical cord plasma (**C**). Values below the limit of detection were imputed as the limit of detection and included in computing the Spearman correlations. The proportion of samples that were below the limit of detection can be seen in Fig. 1.

In maternal plasma, the strongest correlations observed between Th1 and Th2 cytokine concentrations were between TNF-α and IL-12p70 (r = 0.74), IL-4 and IL-12p70 (r = 0.66), and IL-12p70 and IL-10 (r = 0.66, Fig. 5). In umbilical cord plasma, the strongest correlations observed were between IL-12p70 and IL-10 (r = 0.65), IL-5 and IL-2 (r = 0.65), and IL-12p70 and IL-5 (r = 0.64) (Fig. 5). Correlations between cytokines measured in paired maternal and umbilical cord plasma samples were weak, with TNF-$\mathrm{\alpha }$ having the highest correlation of 0.18 among Th1 and Th2 cytokines.

### Partial least squares discriminant analysis (PLSDA)

**Figure 6:**
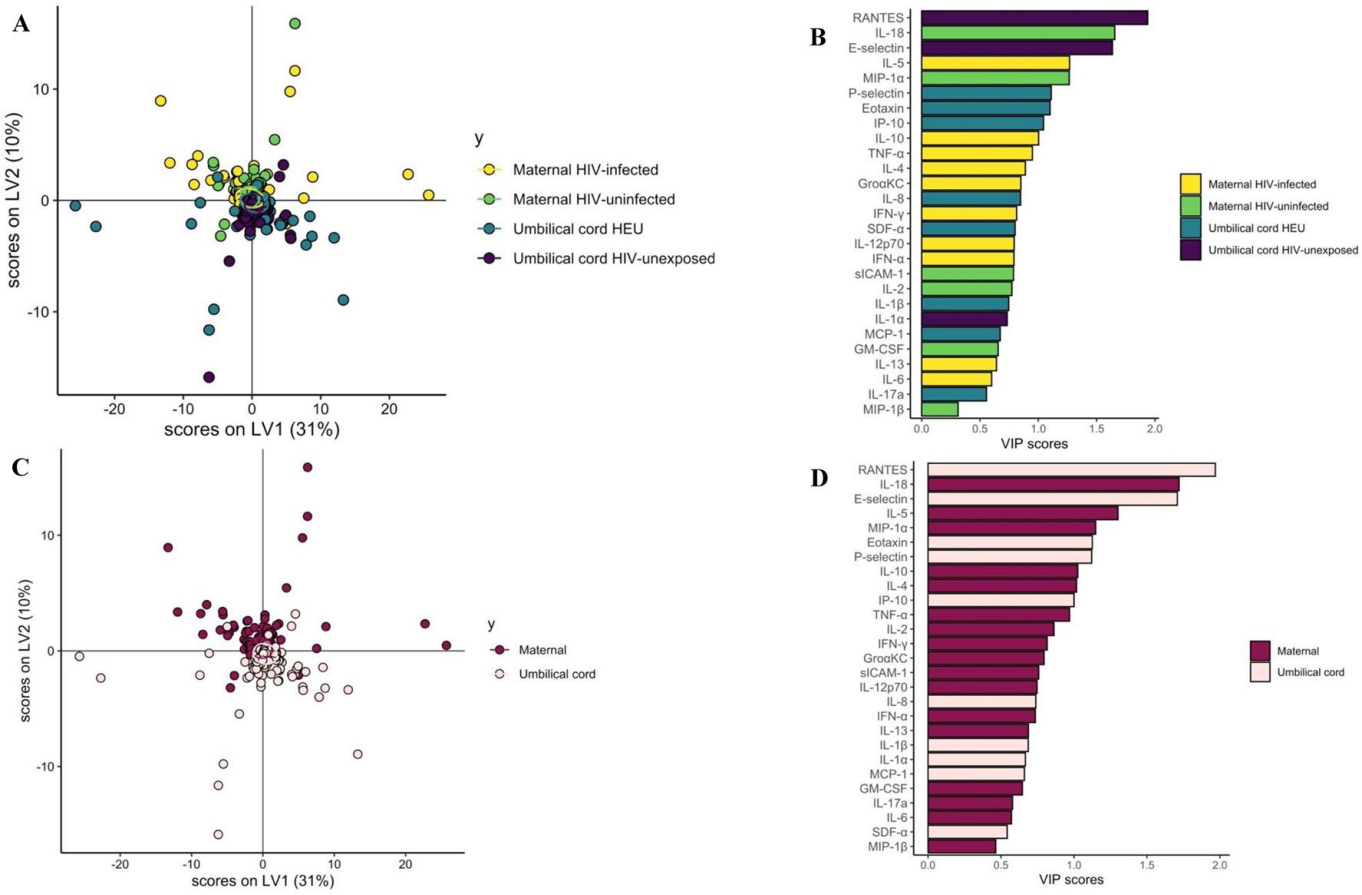
Partial Least Squares Discriminant Analysis (PLSDA) demonstrating significant separation between cytokine profiles. A and B by maternal and umbilical cord plasma by maternal HIV status (**A** and **B**, *P* < 0.01) and significant separation between cytokine profiles of maternal and umbilical cord plasma (**C** and **D**, *P* < 0.01).

**Figure 7:**
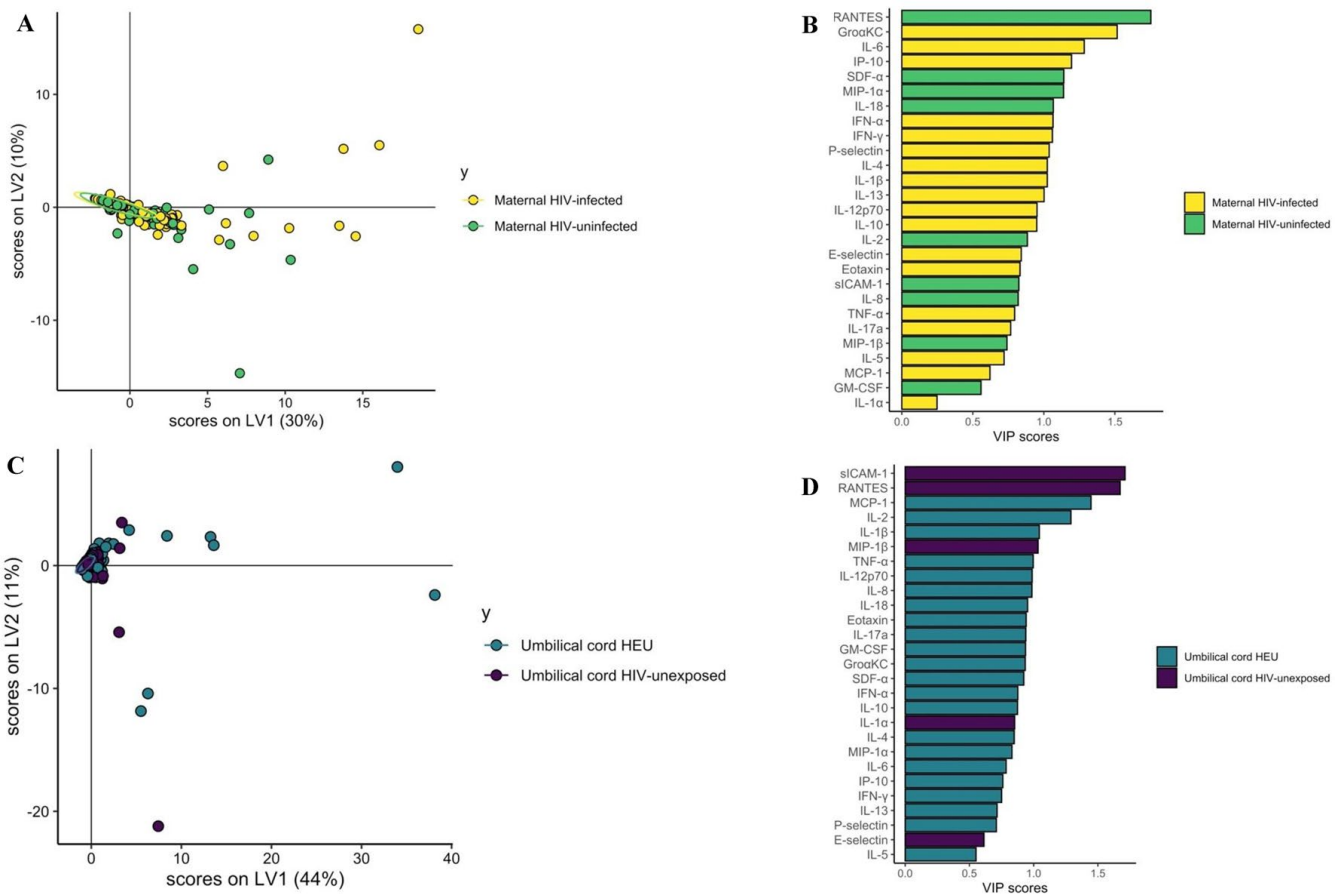
Partial Least Squares Discriminant Analysis (PLSDA) demonstrating significant separation between cytokine profiles of PPHIV and HIV-uninfected women (*P* = 0.02, **A** and **B**) and lack of significant separation between cytokine profiles of HIV-exposed and HIV-unexposed umbilical cord samples (*P* = 0.10, **C** and **D**).

Partial Least Squares Discriminant Analysis (PLSDA) was used to discriminate cytokine profiles between PPHIV, HIV-uninfected pregnant people, HIV-exposed and HIV-unexposed umbilical cord plasma samples (Fig. 6A,B)^27^. RANTES, IL-18, E-Selectin, IL-5, and MIP-1α were the top features distinguishing these four groups (Fig. 7B). The model performed well, with a cross-validation (CV) *P*-value < 0.01.

Maternal and umbilical cord cytokine profiles were also significantly different, with CV *P* < 0.01 (Fig. 6C,D), with higher RANTES and E-Selectin levels in maternal plasma and higher IL-18, IL-5, and MIP-1α levels in umbilical cord plasma (Fig. 6D).

Maternal plasma from PPHIV and HIV-uninfected pregnant people was also significantly different in the PLSDA analysis (Fig. 7A,B), with top features distinguishing the groups being higher RANTES and SDF-α in the HIV-uninfected group and higher gro $\mathrm{\alpha }$-KC, IL-6, and IP-10 in the PPHIV group (Fig. 7B, CV *P* = 0.02). However, a PLSDA model comparing cytokine profiles between HIV-exposed and HIV-unexposed umbilical cord plasma did not demonstrate significant separation between umbilical cord sero-exposure groups (Fig. 7C,D, CV *P* = 0.10).

Overall, our results revealed that cytokine features were distinct between maternal and umbilical cord plasma, PPHIV and HIV-uninfected groups, and differed by maternal HIV viral load detectability. Cytokine features did not appreciably differ between umbilical cord HIV sero-exposure groups, suggesting that HIV infection is associated with a specific maternal peripheral plasma cytokine profile that is not transferred across the placenta. These findings indicate a role for the placenta in coordinating and moderating local inflammatory response.

## Discussion

In this study, we conducted a comprehensive analysis of cytokine profiles during labor in a unique population consisting of 147 PPHIV and 142 HIV-uninfected pregnant people from Uganda, a resource-limited setting. To the best of our knowledge, this is among the first manuscripts to characterize cytokine profiles in this population.

While the overall distribution of cytokine concentrations in both maternal and umbilical cord plasma was similar across different HIV serostatus and viral load groups, we observed a higher proportion of PPHIV with HIV viral load who had elevated levels of maternal plasma IL-6 compared to PPHIV with undetectable viral load and HIV-uninfected participants. IL-6 is a pro-inflammatory cytokine associated with systemic and fetal inflammatory response syndromes (SIRS and FIRS)^28^. Interestingly, a previous study showed higher production of IL-6 in stimulated placental blood cells from PPHIV compared to HIV-uninfected individuals^29^. Additionally, we found elevated levels of IL-10 and IP-10 among PPHIV with detectable HIV viral load. IP-10 is both a chemokine and a potentiator of HIV replication, a classic marker of viral RNA stimulation of immune cells^23^. Previous studies have demonstrated increased IP-10 detection throughout pregnancy in PPHIV compared to HIV-uninfected pregnant individuals^30^. Moreover, elevated placental IP-10 has been associated with a three-fold increase in the risk of in utero HIV transmission^23^. Although IL-10 is considered a net anti-inflammatory cytokine, it has a dual immunologic role with both stimulatory and immunosuppressive effects depending on the environment, and a prior study showed higher levels of IL-10 in the presence of effective ART^20,31^. Collectively, these findings suggest a pro-inflammatory plasma environment in PPHIV with detectable HIV viral load.

Furthermore, we observed a higher proportion of elevated IL-10 concentrations in umbilical cord plasma from PPHIV with detectable HIV viral load compared to those with undetectable viral load and HIV-uninfected individuals. Similarly, umbilical cord plasma samples from PPHIV with HIV viral load exhibited a higher prevalence of elevated MIP-1α and IL-1β concentrations. In a 2007 study, both IL-10 and IL-1β were associated with a placental environment protective against HIV transmission^22^. However, another study from 2013 found lower IL-10 production in plasma from neonates born to PPHIV receiving ART^32^, and a different study of ART regimens around the time of delivery revealed that optimized ART led to greater detectability of placental IL-10, suggesting that elevated IL-10 levels may contribute reduced in utero HIV transmission^24^. MIP-1α and IL-1β are pro-inflammatory cytokines, with MIP-1α playing a crucial role in regulating HIV infection and controlling disease progression^33^. Therefore, the frequent detection of these pro-inflammatory cytokines in umbilical cord plasma from pregnancies with HIV viral load aligns with existing knowledge about cytokine factors that influence HIV transmission risk during pregnancy, and these levels would be expected to be higher with suboptimal suppression of HIV viral load.

Overall, we found weak correlations between cytokine concentrations in maternal and umbilical cord plasma, supporting the lack of active placental regulation of cytokine production in umbilical cord, compared to maternal, blood. However, a 2020 study of cytokine responses in laboratory-stimulated blood samples from Mozambican mother-infant pairs, both with and without HIV, found a positive correlation between maternal and infant responses, suggesting a stronger association between maternal and offspring responses outside of the in utero period^29^. Furthermore, they observed that HIV infection affected the placental blood response, resulting in significantly heightened pro-inflammatory, Th1, and Th17 responses in individuals with HIV who did not receive ART before pregnancy. Since HIV infection is generally characterized by a decline in CD4 T cells, CD8 T-cell expression, and a shift from a Th1 to a Th2 immune response^34^, their findings indicate alterations in innate immune modulation in PPHIV, particularly those who did not undergo ART prior to pregnancy, which could contribute to adverse health outcomes in children.

Using PLSDA models, we discovered distinct patterns of cytokines in the plasma of each group: PPHIV, HIV-uninfected pregnant people, HIV-exposed and HIV-unexposed umbilical cord blood plasma. This separation was driven by higher levels of RANTES in HIV-unexposed umbilical cord plasma, higher levels of IL-8 in HIV-uninfected maternal plasma, higher levels of E-selectin in HIV-unexposed umbilical cord plasma, higher levels of IL-5 in PPHIV plasma, and higher levels of MIP-1α in HIV-uninfected maternal plasma. Although the classical paradigm of Th1 and Th2 responses is overly simplified, IL-8 has been classically associated with a Th1 immune response, while IL-5 is associated with a Th2 immune response. Prior studies have demonstrated that PPHIV exhibit higher frequencies of Th1 cytokines (IL-12, IL-12p70) and the Th2 cytokine IL-5, throughout pregnancy^30^. During labor, the body experiences a pro-inflammatory state, potentially leading to a reversal in immune profile from a Th2-supported state in pregnancy to a Th1 state. However, some studies indicate that the Th2-predominant environment persists throughout labor and only returns to a nonpregnant balance about a week after giving birth^35^. Thus, our findings suggest that HIV-uninfected individuals in labor may exhibit a shift back towards a Th1 response, while PPHIV in our study may not have fully completed this shift.

Our PLSDA findings indicate that there are significant residual differences in cytokine profiles between PPHIV, particularly those with detectable HIV viral load, and HIV-uninfected pregnant individuals, despite receiving combination ART (antiretroviral therapy) with robust CD4 T-cell reconstitution. However, these differences do not extend to umbilical cord plasma, suggesting that the altered maternal immune inflammatory profile may indicate incomplete immune reconstitution, T-cell dysfunction, or ongoing chronic inflammation in PPHIV, especially those with detectable HIV viral load^36,37^. It is important to note that even though all enrolled PPHIV reported taking ART, 28% had detectable HIV viral load, which could be attributed to resistant virus or nonadherence to ART. This highlights the need for further research to determine contributors to ongoing inflammation in PPHIV taking otherwise-effective ART.

Our study differs from previous research in characterizing cytokine profiles in PPHIV in several important ways. One major strength of our study is our large cohort size, which is significantly larger than most prior studies and bolsters confidence in our findings, particularly the differences observed between maternal HIV serogroups in the PLSDA analysis. Another distinguishing feature is that we focused specifically on cytokines during labor, a crucial but understudied phase of pregnancy. Although the acute stress of labor and delivery may alter maternal and umbilical cord plasma cytokine measurements, as a result, our study makes a unique contribution to the literature. An additional strength is that our study encompassed both HIV-infected/exposed and HIV-uninfected/unexposed participants from the same resource-limited setting, an under-studied population representative of a large proportion of PPHIV. Importantly, our enrollment occurred during the era of universal ART with modern regimens, and we utilized actual blood samples made into plasma rather than stimulated cells, further enhancing the relevance and reliability of our analysis.

There are, however, several important limitations to consider. While we collected and compared samples from different blood compartments, it is worth noting that cytokines in umbilical cord plasma could originate from the mother, placenta, or fetus and the type of cells from which the cytokines were secreted is also unknown^38^. In normal term pregnancies, IL-10 found in the bloodstream is thought to originate from the placenta, while IL-6 and IL-8, derived from the fetus, increase during labor and delivery^39^, regulated by the placenta in a non-saturable manner. Thus, we might expect some correlation between maternal and umbilical cord cytokine concentrations. Despite this, we found minimal correlation on a per-cytokine basis. While previous studies have identified correlations between maternal and infant cytokine levels, the relationship between maternal and umbilical cord samples remains poorly characterized^29^ and relationships between cytokine profiles in maternal peripheral blood, placental blood, and umbilical cord blood remain unclear^40^. Additionally, we collected limited data on maternal coinfections and inflammatory disorders that could potentially influence maternal cytokine production. Although we describe differences in Th1 and Th2 responses, it is important to note that T helper responses are not entirely discrete. There is considerable overlap and crosstalk between Th1 and Th2 responses, limiting the utility of this dichotomy^41^. Unfortunately, we were also unable to measure cytokines in the placental blood compartment^29^.

Overall, there has been a significant gap in understanding the overall cytokine landscape during pregnancy. The impact of maternal comorbidities, including chronic infections like HIV, have been largely unexplored. Our study provides new evidence for ongoing maternal inflammation in PPHIV despite ART, especially in PPHIV with detectable HIV viral load. It is well established that immune activity during gestation is linked to long-term child health outcomes, including disrupted immune responses, impaired growth, increased susceptibility to infections, and impaired cognitive ability and executive function^28,42–46^. Future studies of the cytokine environment in HIV-affected pregnancies should thus investigate the relationship between cytokine profiles and possible regulators of the immune and inflammatory environment in pregnancy, including placental histology, transplacental antibody transfer, and impacts on birth and child health outcomes. Additionally, studying the epigenetic profiles of immune cells would be particularly relevant to better understanding the long-term consequences in HIV exposed uninfected neonates, and exploring the influence of specific maternal antiretroviral regimens on inflammatory cytokine profiles in pregnancy would provide further insights to help optimize maternal ART regimens and improve both maternal and fetal health.

## Methods

### Study site, participant recruitment, data collection, and ethical approval

A prospective cohort of 289 pregnant participants was enrolled at Mbarara Regional Referral Hospital (MRRH) in Mbarara, Uganda between September 2017 and February 2018. MRRH serves a mixed urban–rural population of nine million people and reports approximately 11,000 deliveries annually. All pregnant people presenting to MRRH for delivery were screened for enrollment. Pregnant people were eligible if the potential participant was at least 18 years of age, spoke English or Runyankole (the local language) well enough to provide written informed consent, and were available by phone for post-discharge contact. Exclusion criteria included a known or suspected multiple gestation pregnancy, or inability to collect and sample the participant’s placenta. PPHIV who reported not taking combination antiretroviral therapy (ART) were also excluded. Every eligible PPHIV was enrolled, and the next eligible pregnant person without HIV was also enrolled. After enrolling 150 participants, a parity criterion was added for pregnant people without HIV to balance parity by HIV serostatus. HIV-uninfected participants were not otherwise matched to people with HIV. Gestational age was defined by participant report of last normal menstrual period, or chart documentation of last normal menstrual period if participant report was missing. Details of HIV-specific care were obtained by contacting the clinic where each PPHIV received their care to ensure ART accuracy, with participant consent.

Cytokine measurement in maternal and umbilical cord blood was a pre-planned analysis. A structured interview and chart review were performed with each participant to gather demographic, obstetric, and medical details. Data were entered into a Research Electronic Capture (REDCap) database by research assistants working in Uganda^47^. The study was conducted in accordance with the ethical standards laid down in the 1964 Declaration of Helsinki and its later amendments and was approved by the institutional ethics review boards at Mbarara University of Science and Technology (11/03-17), Partners Healthcare (2017P001319), and the Uganda National Council of Science and Technology (HS/2255). No samples or data were collected from prisoners, and all participants provided informed consent to participate prior to sample and data collection.

### Sample collection

Blood was collected from pregnant participants during labor via peripheral venipuncture, and HIV viral load and CD4 + T-cell count measurements were performed for PPHIV if results of these tests were not available within the last 6 months. If more than one HIV viral load or CD4 + T-cell count was measured during the 6 months prior to delivery, the most recent one was used in this analysis. HIV viral load was detectable at a level of 40 or 200 copies/mL, depending on testing location. Umbilical cord blood was collected from the umbilical vein within 30 min of placental delivery. Blood was centrifuged at 3000 g for 20 min to obtain plasma, which was transferred using a micropipette to cryovials for storage at −80 °C.

### Cytokine measurement

ProcartaPlex™ Multiplex Immunoassays (Invitrogen, EPX200-12185-901, EPX200-12173-901) were combined and used to measure 27 chemokines and inflammatory cytokines for 289 participants in both maternal and umbilical cord plasma samples according to the manufacturer’s instructions. There was insufficient reagent to analyze samples for 60 dyads and 3 dyads were excluded due to missing umbilical cord blood samples.

Measured cytokines and chemokines included IL-1β, IL-2, IL-4, IL-5, IL-6, IL-8, IL-10, IL-12p70, IL-13, IL-17A, IL-18, MIP-1α, granulocyte–macrophage colony-stimulating factor (GM-CSF), TNF-α, P-selectin, IL1-α, soluble intracellular adhesion molecule (sICAM)-1, E-selectin, IFN-γ, MCP-1, IFN-α, MIP-1β, SDF-α, Eotaxin, RANTES, and GROα/KC. First, plasma samples were diluted 1:1 in assay buffer and combined with 50µL/well pre-coated beads in each well for a final sample dilution of 1:4. Samples were incubated for 2 h at room temperature, shaking at 500 rpm. Unbound cytokines were removed using an automated plate washer and 25 µL/well of detection antibody was added, followed by a 120-min incubation shaking at room temperature. Then, 12.5 µL Streptavidin-PE was added per well and incubated for another 30 min shaking at room temperature. Excess secondary antibody was removed by washing, and beads were resuspended 50µL/well of Qsol buffer and read on an Intellicyt iQue (Sartorius, Göttingen, Germany) system. Four-fold dilution standards (1:100 to 1:1600) were performed in triplicate to quantify cytokine concentrations.

### Interpolating cytokine concentrations from standard curves

Triplicate raw optical density (OD) values from dilution standards were used to interpolate cytokine concentrations in samples using a sigmoidal five parameter logistic (5PL) curve (Supplementary Fig. 1)^48^.

### Statistical analysis

Cytokine concentrations were natural log-transformed due to skewed distributions and the presence of some extreme outliers. To assess whether PPHIV tend to have extremely high or low concentrations for certain cytokines, we dichotomized each cytokine at the 90th percentile and computed the proportion of pregnant people with a cytokine concentration > 90th percentile for each cytokine. Summary statistics were used to summarize cytokine results separately for PPHIV and for HIV-uninfected participants. Fisher’s exact test was used to compare the proportion of people with an extreme cytokine concentration between the groups HIV-uninfected, PPHIV with undetectable HIV viral loads, and PPHIV with detectable viral loads. McNemar’s test was used to compare the proportion of detectable samples for each cytokine between maternal and umbilical cord pairs. Spearman correlations were used to measure associations between cytokine concentrations.

To further describe the cytokine environment during labor in PPHIV and HIV-uninfected pregnant people, cytokines were grouped according to phenotype as Th1 (IFN-γ, IL-12p70, IL-2, TNF-α) and Th2 (IL-10, IL-13, IL-4, IL-5). The natural log of cytokine concentrations was summed across Th1 and Th2 categories for maternal and umbilical cord blood separately. The spearman correlation was computed to assess the association between total Th1 and Th2 cytokine concentrations.

#### Partial least squares discriminant analysis (PLSDA)

To visualize how cytokine features differentiate between PPHIV and HIV-uninfected pregnant people, PLSDA models were built to compare groupings by maternal and umbilical cord plasma source, and HIV serostatus^27,49^. PLSDA, derived from principle component analysis, is a statistical multivariate dimension-reduction tool, and supervised machine learning classifier.

One model helped to classify four categories (maternal blood HIV-, maternal blood PPHIV, umbilical cord blood HIV-, umbilical cord blood PPHIV). Three models helped define the class label in two categories (maternal blood or umbilical cord blood; and HIV- or PPHIV; performed separately for maternal blood and umbilical cord blood samples). Cytokine values were centered and scaled to a standard deviation of 1.0 (z-score). Latent Variable 1 (LV1) captured feature variance in pairwise group separation, and Latent Variable 2 (LV2) captured variance orthogonal (not contributing) to LV1. Scores and loadings were visualized to identify cytokine features accounting for the greatest variance. A permutation (100 permutations) test was performed, and fivefold cross validation (CV) was performed. Variable Importance in Projection (VIP) scores were calculated^50^ and ordered to rank the importance of each cytokine used in each PLSDA model, and then plotted according to the direction and group within which they were statistically significantly enriched.

All statistical analyses were conducted in R version 4.2.2^51^, and figures were created using the ggplot2 package^52^. With 85 statistical tests conducted—27 associated with Fig. 1, 54 associated with Fig. 3, and four associated with the PLSDA—a Bonferroni-adjusted significance level of 0.00059 conservatively preserves a 5% type I error rate.

### Supplementary Information

Supplementary Figures.

## Data Availability

Data collected for the study, including individual participant data and a data dictionary defining each field in the set, will be made available to others as deidentified participant data from the investigators on request after publication. The study protocol will also be available on request after publication. Those requesting access to data should email LMB, corresponding author, to create a data access agreement, which must be fully executed before any study data or materials can be shared.
