# Supplementary material for: Distinct cytokine profiles in late pregnancy in Ugandan people with HIV
Source: Sci Rep. 2024 May 14;14:10980. doi: 10.1038/s41598-024-61764-2 (PMC11093984; doi:10.1038/s41598-024-61764-2)

**Supplementary Information**

| **Supplementary Figure 1**. Cytokine standard curves, used to interpolate cytokine concentration values in pg/mL from optical density values. Cord = umbilical cord, Mat = maternal, Conc = concentration. | Pg. 2 |
| --- | --- |
|  |  |
| **Supplementary Figure 2**. Cytokine concentration for **other cytokines** **with high levels of detection** in maternal and umbilical cord plasma. Cytokine concentrations were natural log transformed. | Pg. 3 |
|  |  |
| **Supplementary Figure 3**. Cytokine concentration for **other cytokines with moderate levels of detection** in maternal and umbilical cord plasma. Cytokine concentrations were natural log transformed. | Pg. 4 |
|  |  |
| **Supplementary Figure 4**. Cytokine concentration for **other cytokines with low levels of detection** in maternal and umbilical cord plasma. Cytokine concentrations were natural log transformed. | Pg. 5 |
|  |  |
| **Supplementary Figure 5.** The fold-change of maternal cytokines with respect to umbilical cord cytokines. Cytokine concentrations were natural log transformed before computing the ratio of maternal:cord | Pg. 6 |
|  |  |
|  |  |
|  |  |

Supplementary Figure 1. Cytokine standard curves, used to interpolate cytokine concentration values in pg/mL from optical density values. Cord = umbilical cord, Mat = maternal, Conc = concentration.
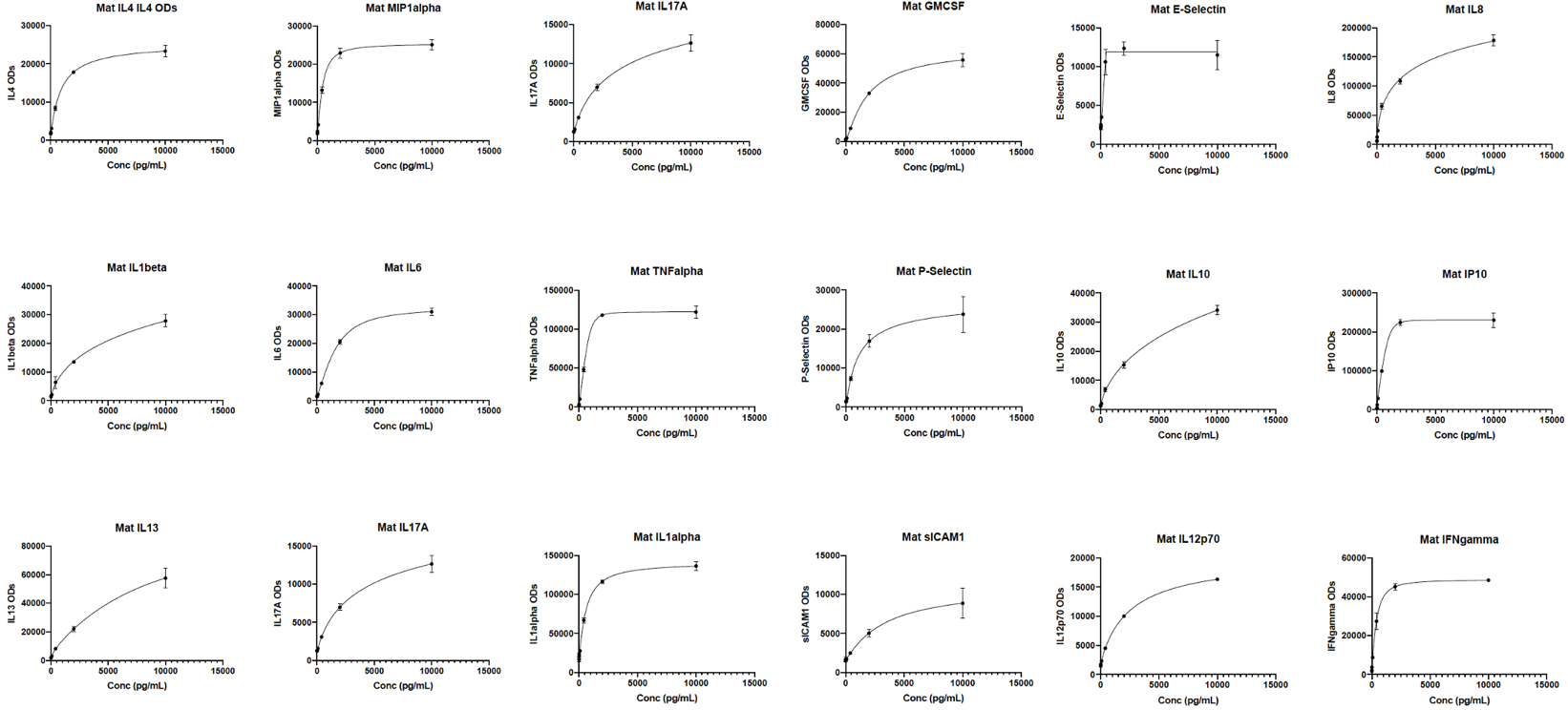


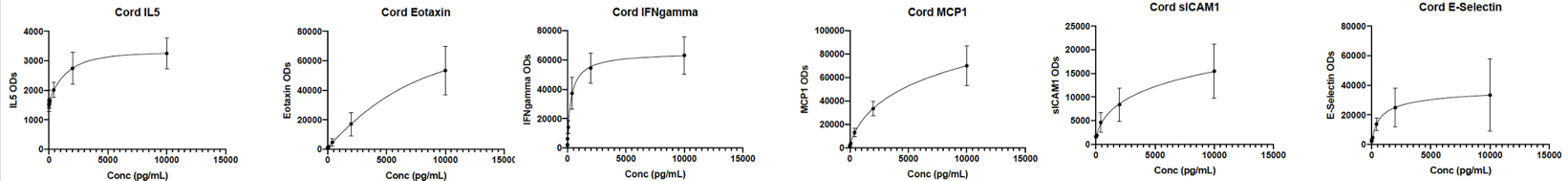


Supplementary Figure 2. Cytokine concentration for **other cytokines with high levels of detection** in maternal and umbilical cord plasma. Cytokine concentrations were natural log transformed.**
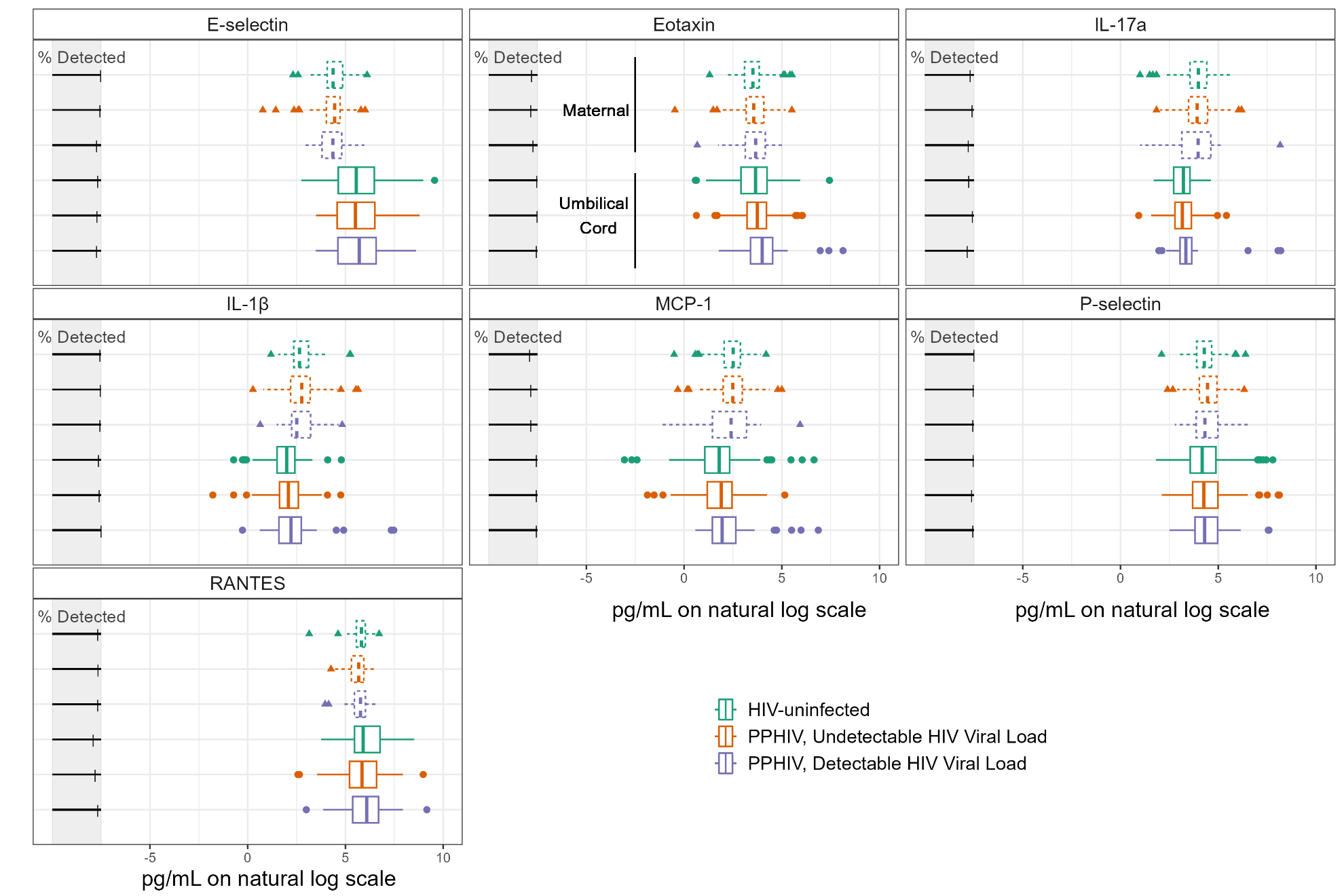
**

Supplementary Figure 3. Cytokine concentration for **other cytokines with moderate levels of detection** in maternal and umbilical cord plasma. Cytokine concentrations were natural log transformed.^
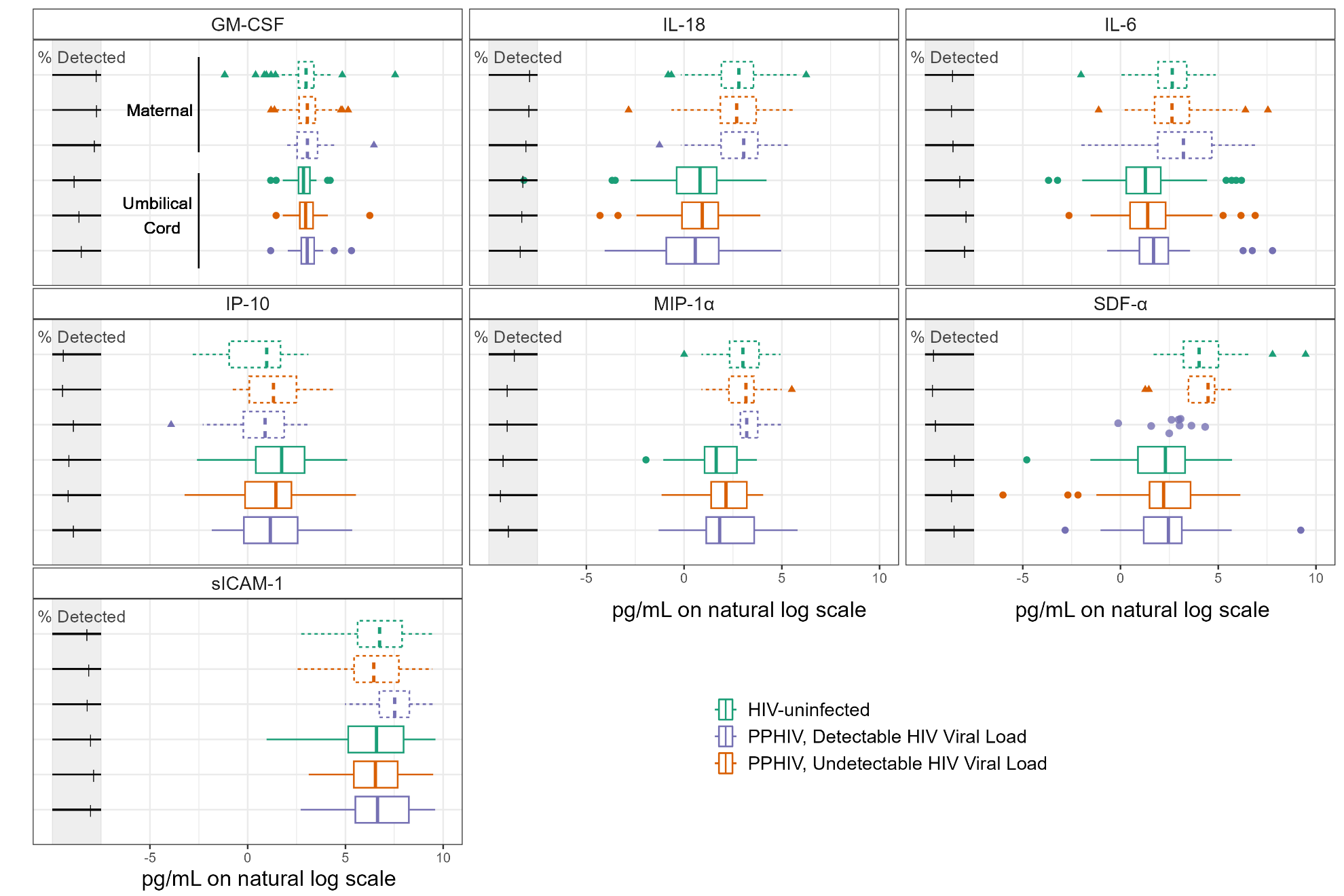
^

Supplementary Figure 4. Cytokine concentration for **other cytokines with low levels of detection** in maternal and umbilical cord plasma. Cytokine concentrations were natural log transformed.^
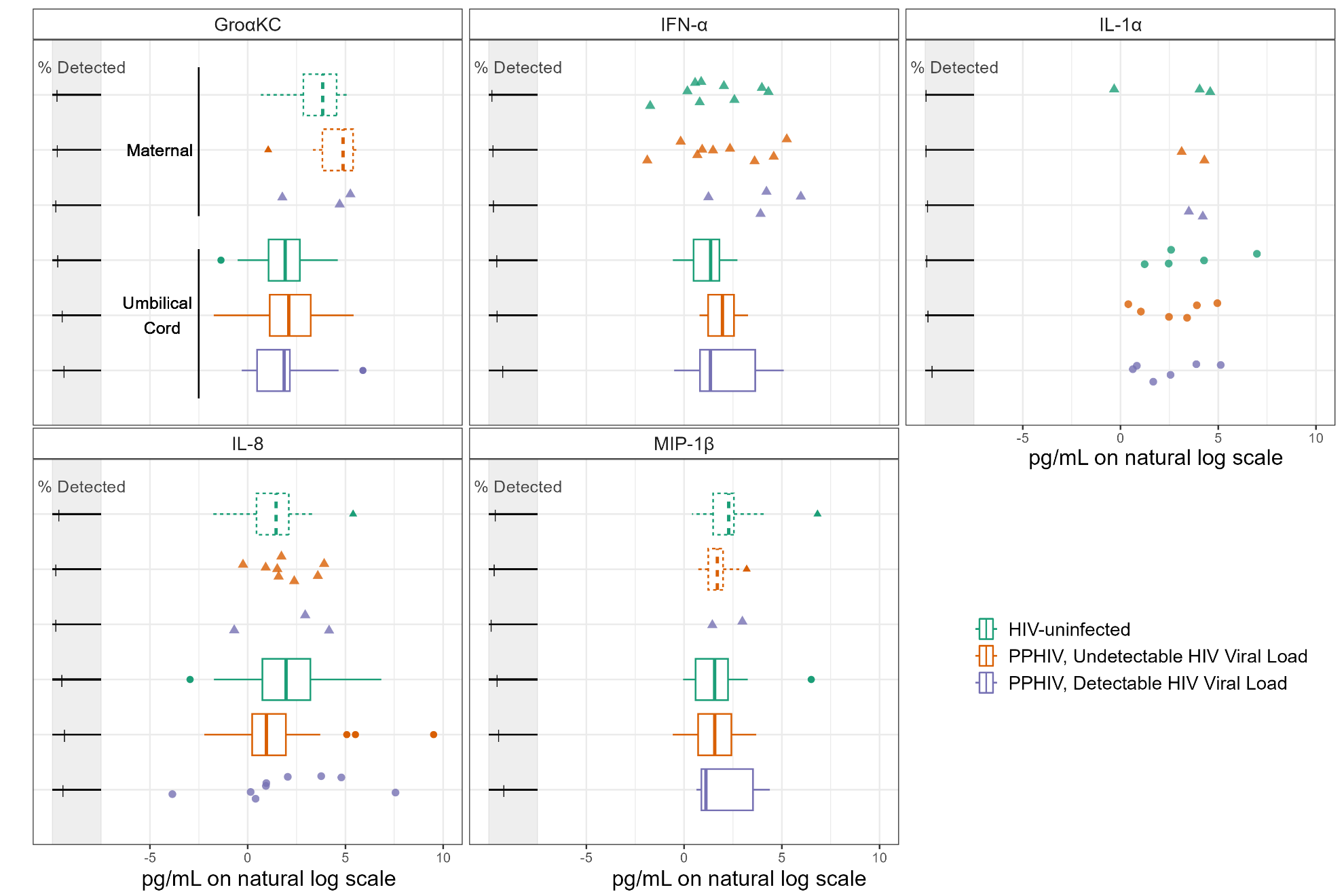
^

Supplementary Figure 5. The fold-change of maternal cytokines with respect to umbilical cord cytokines. Cytokine concentrations were natural log transformed before computing the ratio of maternal:cord.
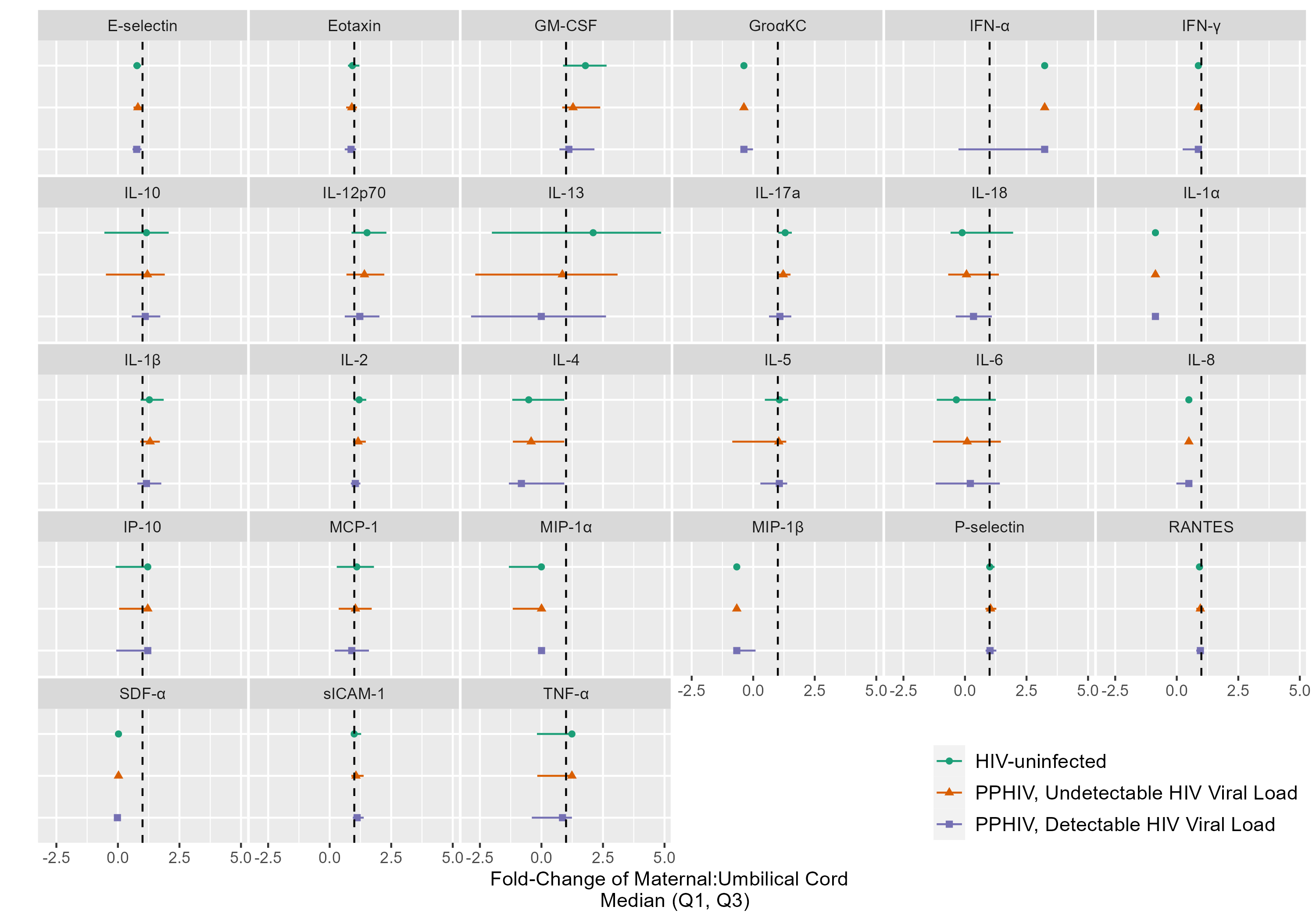

Supplement: Supplementary file 1 — Supplementary Figures. [file 41598_2024_61764_MOESM1_ESM.docx]
